# Protective effects and mechanisms of the Erzhi formula on glucocorticoid induced primary cortical neuron injury

**DOI:** 10.3389/fphar.2023.1038492

**Published:** 2023-02-27

**Authors:** Rui Han, Guoying Han, Yiqi Yan, Lifeng Han, Lin Li, Han Zhang

**Affiliations:** ^1^ Institute of Traditional Chinese Medicine, Tianjin University of Traditional Chinese Medicine, Tianjin, China; ^2^ Key Laboratory of Pharmacology of Traditional Chinese Medical Formulae, Tianjin University of Traditional Chinese Medicine, Ministry of Education, Tianjin, China; ^3^ State Key Laboratory of Component-Based Chinese Medicine, Tianjin University of Traditional Chinese Medicine, Tianjin, China

**Keywords:** the erzhi formula, primary cortical neuron, stress, depression, glucocorticoids, apoptosis, synaptic plasticity, network analysis

## Abstract

High concentrations of glucocorticoids (GC) can cross the blood-brain barrier into the brain parenchyma, triggering a stress state that can lead to a range of physiological changes. This study investigated whether Erzhi formula has neuroprotective effects against glucocorticoid damage by establishing a dexamethasone-induced primary cortical neuron injury model *in vitro*. The results showed that Erzhi formula could reduce dexamethasone-induced apoptosis in primary cultured cortical neurons and improve synaptic damage. Further, network pharmacological analysis revealed that Erzhi formula may exert antidepressant effects by multi-component, multi-target, and multi-pathway characteristics, in which Salidroside, Biochanin-A and other ingredients are key components, HSD11B1, NR3C1, and other proteins are key targets, and steroid metabolism may be a key process in its action. Moreover, our study found that the neuroprotective effect of Erzhi formula might be related to the 11β-HSD1-GC/glucocorticoid receptor (GR) signaling pathway. The Erzhi formula could significantly inhibit the activity of 11β-hydroxysteroid dehydrogenase 1 (11β-HSD1) *in vitro* using homogeneous time-resolved fluorescence. In addition to providing evidence for the pharmacological effects of the Erzhi formula, the present study lays down the foundation for subsequent experiments.

## 1 Introduction

Depression is a major emerging mental mood disorder worldwide, with excessive stress being one of its primary causes. Prolonged stimulation can cause dysfunction of the sympathetic-adrenomedullary system and the hypothalamic-pituitary-adrenal (HPA AXIS), leading to neural network remodeling and neuronal damage, or even psychiatric disorders such as depression or cognitive impairment (Zhang et al., 2020). HPA AXIS dysfunction can lead to abnormal corticosterone regulation, impaired neurogenesis, synaptic dysfunction, and other problems that can further lead to depressive-like behavior.

The glucocorticoid (GC) system plays an important role in the regulation of the HPA AXIS, and endogenous cortisol is an important stress hormone. Studies have shown that elevated cortisol is beneficial to the body’s response to external stimuli and stress; however, the high concentrations of cortisol produced under prolonged uninterrupted stress not only jeopardize cardiovascular system function, but also cause dendritic atrophy of neurons, synaptic structure change, and neuronal apoptosis, which ultimately induces cognitive dysfunction (Ajala, 2017). In addition, exogenous GC may increase the risk of neurodegenerative diseases by promoting lactate dehydrogenase (LDH) release from hippocampal neurons and altering neuronal synaptic structure and function (Macpherson et al., 2005; Finsterwald et al., 2015). Sustained stress causes hyperactivity of the HPA AXIS, resulting in excessive GC secretion. After crossing the blood-brain barrier to enter the hippocampus or cortical tissues, GC levels in local tissues are regulated by the negative feedback effect of the glucocorticoid receptor (GR) (Terunuma et al., 2003). In contrast, mifepristone (RU486), a GR blocker, may improve cognitive impairment by blocking synaptic damage to hippocampal neurons induced by stressful doses of GC (Block et al., 2017). 11β-hydroxysteroid dehydrogenase 1 (11β-HSD1) is a key enzyme in the intracellular production of cortisol, is widely distributed in the liver, brain, and adipose tissues, and plays a dual role in redox and dehydrogenation, regulating the conversion between non-biologically active cortisol (human)/11-dehydrocorticosterone (mouse) and active cortisol (human)/corticosterone (CORT) (mouse) (White, 2018). Studies have shown that RU486 completely inhibits the upregulation of 11β-HSD1 mRNA expression induced by the stress hormone cortisol (Wyrwoll et al., 2011) and inhibition of 11β-HSD1-induced reduction in GC is strongly associated with improved cognitive performance (Puigoriol-Illamola et al., 2020a; Puigoriol-Illamola et al., 2020b). Currently, stress-related diseases are attracting increasing attention. Stress may trigger damage to the body, and in turn, the imbalance or dysfunction caused by the damage may further amplify stress, causing the body to enter a negative cyclic chain.

The Erzhi formula, consisting of Ligustri lucidi fructus and Ecliptae herba, is a classical traditional Chinese medicine (TCM) prescription for nourishing the liver and kidney. It is mostly used clinically for the treatment of liver and kidney yin deficiency, such as dizziness, insomnia and dreaminess, soreness and weakness of the waist and knees, spermatorrhea, and premature gray hair (Lim et al., 2018). Meanwhile, Yin deficiency of liver and kidney is one of the most common syndromes of depression, suggesting that attention should be paid to liver and kidney tonifying in the clinical treatment of depression. Pharmacological studies have shown that Erzhi Pill can improve the learning and memory ability of aging rats, scavenge free radicals, protect neural function and show anti-aging effects (Wang et al., 2017). A previous study found that the Erzhi formula significantly reduced CORT levels and downregulated CRH mRNA, adrenal CORT synthase 21α-hydroxylase (CYP21A1), and steroid acute regulatory protein (STAR) mRNA in the cortex and hypothalamus of mice under a chronic mild unpredictable stimulus model. These results suggested that the Erzhi formula can inhibit restraint stress-induced hyperactivation of the HPA AXIS from both central and peripheral sources.

In our study, we established a model of dexamethasone-induced injury in primary cultured cortical neurons at stress doses and investigated the function and mechanism of the Erzhi formula in ameliorating neuronal injury using various methods and network pharmacological analysis.

## 2 Materials and methods

### 2.1 Drug and chemicals

Dexamethasone (DEX), mifepristone (RU486), and carbenoxolone disodium salt (CBX) were purchased from Sigma, United States. The Erzhi formula was provided by the Department of Pharmacy, Institute of Traditional Chinese Medicine, Tianjin University of Traditional Chinese Medicine. The Erzhi formula was prepared by mixing the alcoholic extract of Ligustrum lucidum Ait (Oleaceae Hoffmanns; Ligustri lucidi fructus) and Eclipta prostrata L (Asteraceae Bercht; Ecliptae herba) in a 1:1 ratio with raw herbs. Ligustri lucidi fructus and Ecliptae herba were purchased from the Beijing Tongrentang Group Tianjin Ping Shan Dao Pharmacy Co. Both Ligustri lucidi fructus and Ecliptae herba were extracted by heating and refluxing with 70% ethanol; the procedure was performed three times, each time for 1 h, and the amount of 70% ethanol was increased by 10 times of raw herbs for each cycle. Then the extracts were combined and concentrated in an electric heating set (98-1-C, Tianjin Taisite Instrument Co., China) at 55°C to form a concentrated solution, and the concentrated solution were luophilized with an electric vacuum drying oven (DZG-6050, Shanghai Pein Experimental Instruments Co., China) at 45°C. Finally, 24.29 g powder was obtained from 100.00 g of Ligustri lucidi fructus and 20.80 g powder was obtained from 100.00 g of Ecliptae herba.

### 2.2 Primary cortical neuronal cultures

Cortical neurons were obtained from an embryonic 16-day Wistar rat (Beijing Weitong Lihua Laboratory Animal Technology Co., Ltd.). The cortex was separated, clipped, and digested by trypsin (Gibco, United States). Cells were plated in a 55 mm dish at a density of 1 × 10^6^ cells/mL or in 96-well or 24-well plates at a density of 5 × 10^5^ cells/mL coated with poly-d-lysine after resuspension in DMEM/F12 basal medium (Gibco, United States) containing 10% FBS (Gibco, United States) and 1% penicillin and streptomycin (Gibco, United States). The cells were maintained in DMEM/F12 basal medium supplemented with 2% B27 (Gibco, United States), 1% penicillin and streptomycin, and 0.5% glutamine (Gibco) after 4 h. In all experiments, the cells were cultured in a cell culture incubator (Thermo Fisher, United States) at 37°C and 5% CO^2^. After 5 days of culture, DEX, Erzhi formula, or other drugs were administered and then cultured for 72 h for subsequent experiments.

### 2.3 MTT

0.5 mg/mL solution of methyl thiazolyl tetrazolium (MTT) (Sigma, United States) was added to the cells cultured in 96-well plates and incubated for 4 h at 37°C in a 5% CO_2_ cell incubator (protected from light). The original culture solution was replaced with dimethyl sulfoxide (DMSO) (Sigma, United States), and the crystals were dissolved by shaking for 10 min on a microtiter (Tianjin Hua Bei Experimental Instruments Co., Ltd.). The optical density (OD) value was measured at 570 nm using an enzyme marker (Tecan, Switzerland).

### 2.4 LDH

The treated cell supernatants harvested from 96-well plates were mixed with lactate dehydrogenase (LDH) working solution (Dojindo, Japan) at a ratio of 1:1 for 30 s on a microplate shaker. After 30 min of incubation at room temperature in a light-protected environment, the stopping solution was added to the plate. The optical density (OD) value was measured at 490 nm using an enzyme marker to evaluate cell viability.

### 2.5 TUNEL

Apoptosis was detected using a TUNEL staining kit (Merck Sharp and Dohme, MSD) according to the manufacturer’s instructions and a previously reported method, and then photographed with an inverted fluorescent microscope.

### 2.6 Western blot

Protein samples were harvested from cortical neurons cultured in a 55 mm dish after treatment. Total proteins were lysed using RIPA protein extraction buffer (Solarbio, Beijing, China) according to the manufacturer’s instructions, and protein concentrations were determined using a BCA Kit (ThermoFisher, United States). Equal amounts of protein aliquots were used to check the expression levels of target proteins using anti-Caspase-3, anti-Cleaved Caspase-3, anti-Bcl-2, anti-Bax, anti-PSD95, anti-SYN, and anti-β-tubulin (CST, United States) (1:1,000) through 10% SDS-PAGE gel electrophoresis. After incubation with the appropriate HRP-conjugated antibody (1:10,000) (Beijing Zhongshanjinqiao Biotechnology Co, Ltd), the proteins were visualized using the ChemiDocTM MP Imaging System (Bio-Rad, United States).

### 2.7 Immunostaining

Cells cultured in 24-well plates were fixed in ice-cold 4% paraformaldehyde (Solarbio, China) for 5 min, washed in PBS (Solarbio, China), incubated in preblock buffer (0.3% Triton X-100 (Solarbio, China) and 5% goat serum (Solarbio, China) in PBS) for 60 min, and then incubated overnight (4°C) with primary antibodies microtubule-associated protein 2 (MAP-2) diluted (1:200) in preblock buffer. After washing with PBS, cells were incubated with secondary antibody Alexa Fluor 488-Goat anti-Rabbit IgG (ThermoFisher, United States) (1:1,000) in preblock buffer at room temperature for 2 h. Next, DAPI (4’,6-diamidimo-2-phenylindole) nuclear staining solution (Solarbio, China) was added and incubated for 10 min at room temperature. After washing with PBS, an anti-fluorescence quenching agent was added (Beyotime Biotechnology, China), and the views were photographed under an inverted fluorescent microscope.

### 2.8 Network analysis

#### 2.8.1 Screening for active ingredients of the Erzhi formula and potential targets prediction

The components of the Erzhi formula that are absorbed into the blood were downloaded from the PubChem database (https://pubchem.ncbi.nlm.nih.gov) and then screened using the “lter by Lipinski and Veber Rules” algorithm of Discovery Studio™ software (i.e., setting the selection criteria to <500 molecular weight, <5 hydrogen bond donors, <10 hydrogen bond acceptors, <5 lipid-water partition coefficients, and ≤10 rotatable bonds) to obtain the active components of the Erzhi formula. The CTD database (https://ctdbase.com), HERB database (http://herb.ac.cn), Swisstarget prediction database (http://swisstargetprediction.ch), and Perl software were used to identify and predict relevant targets of the active ingredients. The information obtained on the active ingredients and the related targets was then collated and the Cytoscape 3.7.1 software was used to create an “active ingredients-target” network diagram.

#### 2.8.2 Target acquisition of the Erzhi formula for depression treatment

The GENECARDS database (https://www.genecards.org) was used to obtain targets of depression. Using R language operations, the overlapping core targets mapped from the active ingredient targets to the depression targets were identified as the potential targets of the Erzhi formula for treating depression.

#### 2.8.3 PPI interaction network construction and analysis

Overlapping core targets were imported into the STRING data analysis platform (https://cn.string-db.org/) for protein-protein interaction (PPI) network analysis. PPIs were constructed based on the strength of the relationships between the targets using Cytoscape 3.7.1 software, and clustering analysis was performed.

#### 2.8.4 GO (gene ontology) enrichment and analysis

The overlapping target names were converted into ENTREZ Gene IDs using the R language and analyzed using the DAVID database (https://david.ncifcrf.gov) to obtain information about GO gene function analysis. *p*-value was used as a reference value for screening, and R language and Bioinformatics (https://www.bioinformatics.com.cn) were used to analyze the related content of gene function enrichment analysis.

### 2.9 Homogenous time-resolved fluorescence

Glycyrrhetinic acid (GA) (10 μL) at 1 μM and 0.1–100 μg/mL of the Erzhi formula were placed in a 96-well transparent plate, and 10 μL of liver microsomes at a concentration of 2 g/L were added to each well, centrifuged at 1,000 rpm for 1 min, and shaken on a microplate shaker for 5 min. Next, 30 μL of CORT and nicotinamide adenine dinucleotide phosphate (NADPH) were added to each well, centrifuged at 1,000 rpm for 1 min, and incubated for 2 h at 37°C in an air bath shaker. Prepared cortisol standard (10 μL) was then added to each well. After incubation, 10 μL from the first transparent plate was pipetted into the corresponding wells of a 96-well white plate. Cortisol acceptor (5 μL) and cortisol donor (5 μL) were added to the wells in a white plate and incubated at room temperature for 2 h. Finally, the fluorescence signal at 665 and 620 nm was detected using an enzyme standard meter, and the fluorescence signal was calculated according to the cortisol standard curve. Cortisol concentration in each group was calculated using the standard curve.

### 2.10 Calcium phosphate-DNA coprecipitation

An appropriate amount of green fluorescent protein (GFP) plasmid was mixed with 75 µL of autoclaved ultrapure water. Next, 87.5 µL of HEPES buffer (280 mM NaCl, 10 mM KCl, 1.5 mM Na_2_HPO_4_, 12 mM D-Glucose, and 50 mM HEPES, pH 7.05) was added and mixed, and finally 10.5 µL of CaCl_2_ was added and incubated for 5 min to form a calcium phosphate-DNA precipitate. After 4 days of culture, the calcium phosphate-DNA precipitate was added dropwise uniformly to the cell culture medium, and additional treatments were carried out after 48 h.

### 2.11 Statistical analyses

All statistical analyses were performed using the GraphPad Prism version 8. One-way ANOVA was used to test whether the means of each experimental group were significantly different, and if the overall *p*-value was <0.05, then multiple comparisons between the experimental groups were tested using Tukey’s *post hoc* analysis.

## 3 Results

### 3.1 The Erzhi formula ameliorates DEX-induced primary cortical neuronal damage

**FIGURE 1:**
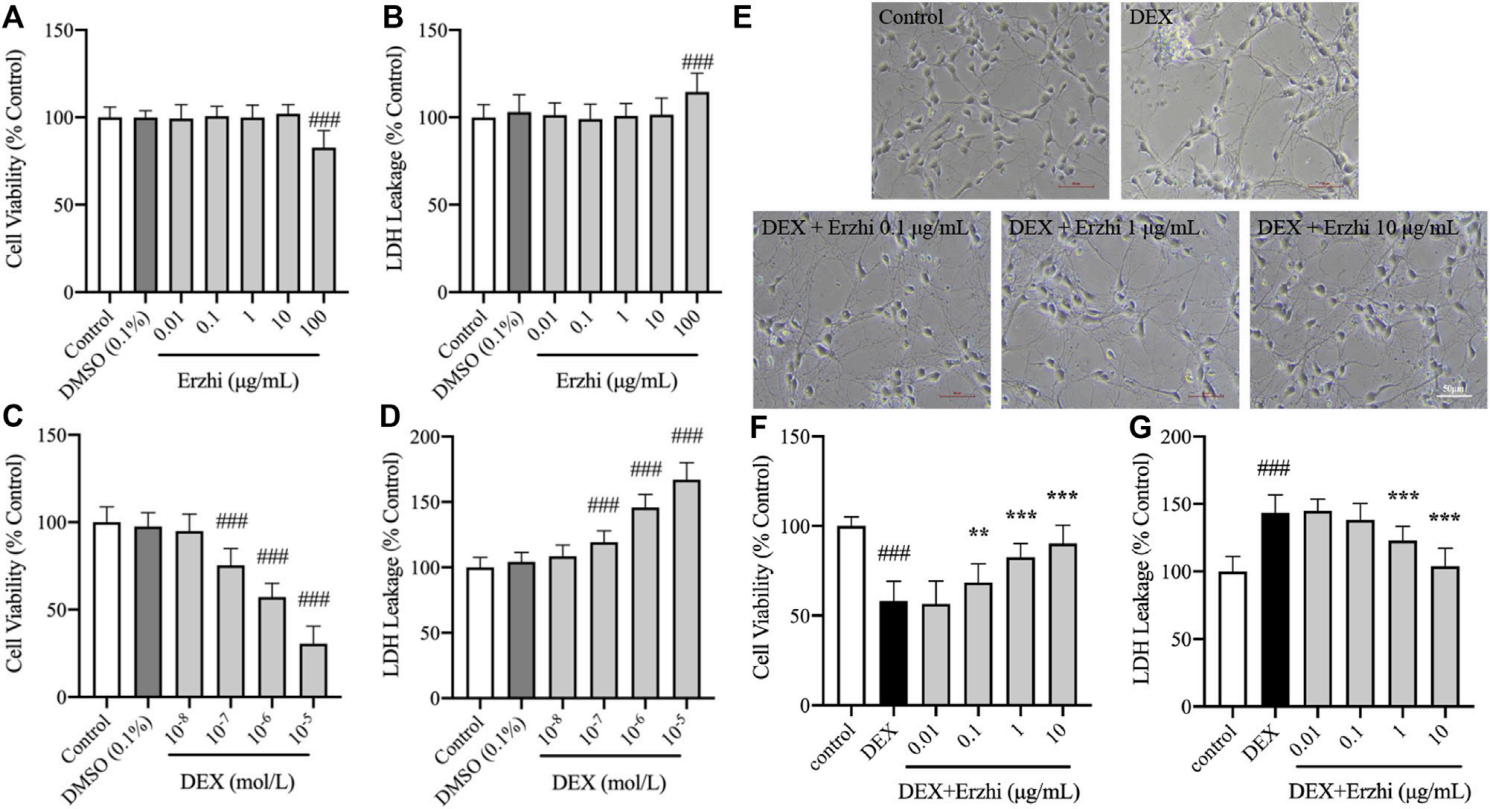
Neuroprotective effects of the Erzhi formula on primary cultured cortical neurons. **(A, B)** Safe dosage selection of Erzhi formula (n = 18 per group). 100 μg/mL Erzhi formula showed significant cell toxicity (*p <* 0.001); **(C, D)** damage concentration selection of DEX (n = 18 per group). 10^−7^ to 10^−5^ mol/L DEX showed cell toxicity; **(E)** growth status of primary cortical neurons with Erzhi formula treatment (bar = 50 μm). 0.1–10 μg/mL Erzhi formula can reverse cell damage; **(F, G)** effective dosage selection of the Erzhi formula (n = 18 per group). The results are expressed as mean ± SD. ^###^ *p* < 0.001 vs. control; ^**^ *p* < 0.01, ^***^ *p* < 0.001 vs. DEX. DEX, dexamethasone; SD, standard deviation.

Compared with the control group, 0.01–10 μg/mL Erzhi formula had no significant toxic effect on primary cultured cortical neurons (Figures 1A, B). However, 100 μg/mL Erzhi formula showed significant cell toxicity (Figures 1A, B), in which cell viability was reduced by approximately 17% and LDH leakage was increased by approximately 14%. Compared with the control group, there was no significant change in cell viability and LDH release in the 10^−8^ mol/L DEX group (*p >* 0.05). Cell viability decreased from 25% to 70% (*p <* 0.001) and LDH release increased from 19% to 67% (*p <* 0.001) in the 10^−7^ to 10^−5^ mol/L DEX groups, respectively (Figures 1C, D). The primary cortical neurons in the 10^−6^ mol/L DEX group showed a decrease in cell viability of approximately 43% and an increase in LDH release of approximately 46%. The 10^−6^ mol/L DEX group was selected for subsequent experiments. Primary cortical neurons cultured for 5 days were more refractive, with round, oval, or irregularly shaped cytosol and two or more protrusions, which were connected to form a net-like structure. However, cells in the DEX group showed wrinkling, broken protrusions, and cell clustering, and some cells showed vacuolation. Further, protrusion breakage and vacuolation of neurons were significantly reduced in the 0.1–10 μg/mL Erzhi formula group (Figure 1E). Compared to the control group, the cell viability of neurons significantly reduced to 55% (*p <* 0.001) and LDH release significantly increased to 145% (*p <* 0.001) after stimulation with 10^−6^ mol/L DEX. There was a significant increase in cell viability (*p <* 0.001) and decrease in LDH release (*p <* 0.001) in the 1–10 μg/mL Erzhi formula group; there was a significant increase in cell viability (*p <* 0.01) and no significant change in LDH release (*p >* 0.05) in the 0.1 μg/mL Erzhi formula group; and finally, there was no significant change in cell viability and LDH release in the 0.01 μg/mL group (*p >* 0.05) (Figures 1F, G). The 10 μg/mL Erzhi formula showed the best protective effect in this group, with cell viability of approximately 90% and LDH leakage of approximately 103%, compared with the control group.

### 3.2 The Erzhi formula ameliorates DEX-induced primary cortical neuronal apoptosis

**FIGURE 2:**
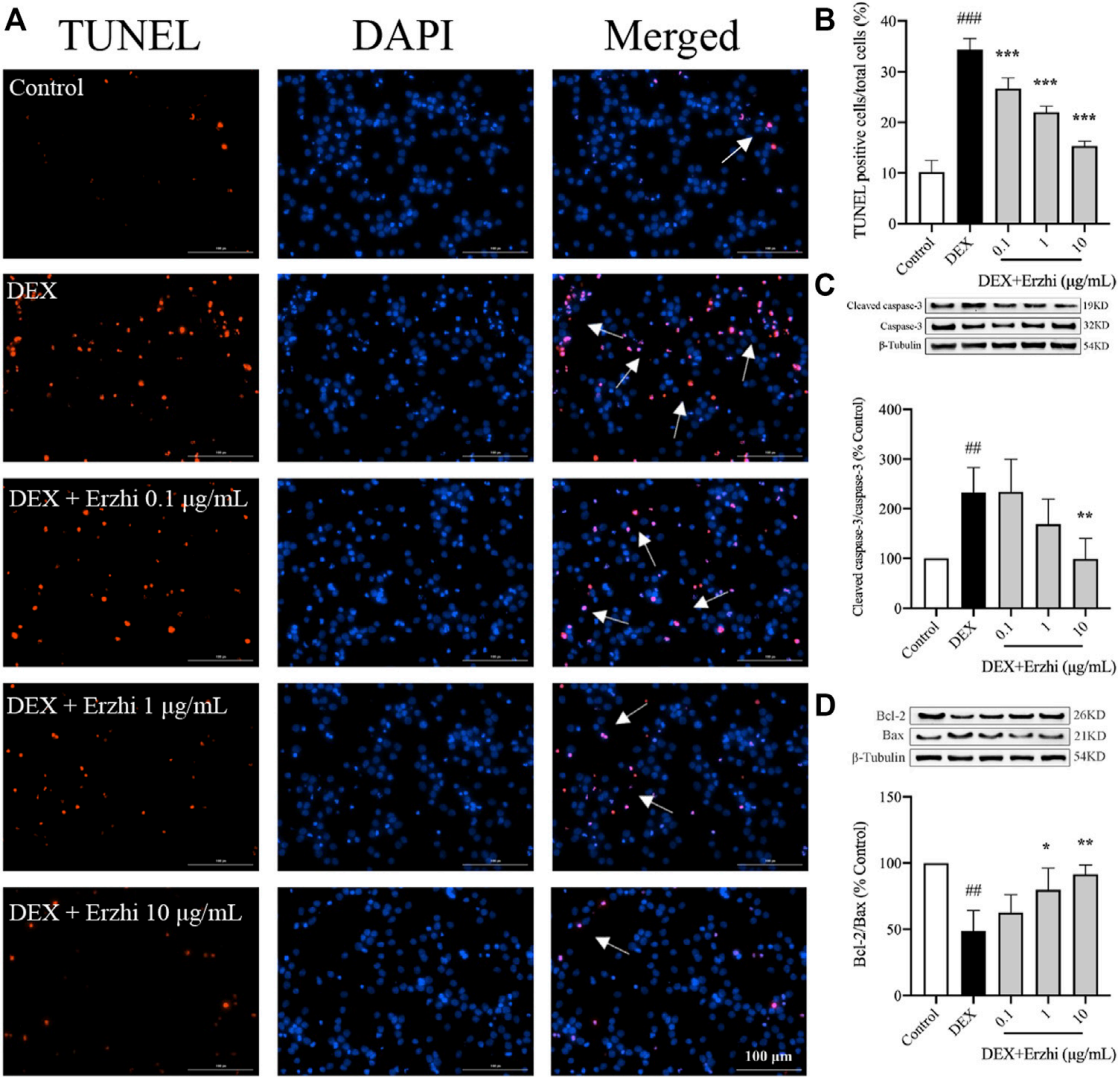
Effect of the Erzhi Formula on apoptosis of primary cortical neurons. **(A, B)** TUNEL fluorescence staining (bar = 100 μm) and quantitative analysis (n = 3 per group). The number of apoptotic cells in the Erzhi formula group was significantly decreased compared with the DEX group (*p <* 0.001); **(C, D)** protein expression of Cleaved caspase-3, Caspase-3, Bcl-2, and Bax (n = 3 per group). The application of the Erzhi formula reversed the dexamethasone-induced increase in Cleaved caspase-3/Caspase-3 expression (*p <* 0.01) and reversed the decrease in Bcl-2/Bax expression (*p <* 0.05). The results are expressed as mean ± SD. ^##^ *p* < 0.01, ^###^ *p* < 0.001 vs. control; ^*^ *p* < 0.05, ^**^ *p* < 0.01, ^***^ *p* < 0.001 vs. DEX.

The results of TUNEL nuclear staining showed that the number of apoptotic cells in primary cortical neurons was significantly increased in the DEX group compared with the control group (*p <* 0.001), and the number of apoptotic cells in the Erzhi formula group was significantly lower than that in the DEX group (*p <* 0.001), with the most significant decrease observed in the 10 μg/mL Erzhi formula group (Figures 2A, B). Compared to the control group, the number of apoptotic cells was approximately 3.4 times higher after stimulating with 10^−6^ mol/L DEX; however, after the application of the Erzhi formula, the number of apoptotic cells reduced by a factor of 1.5 times. Compared with the control group, the expression of Cleaved caspase-3/Caspase-3 was upregulated 2-fold (*p <* 0.01) (Figure 2C) and the expression of Bcl-2/Bax was downregulated to half of the original in the DEX group (*p <* 0.001) (Figure 2D). The expression of Cleaved caspase-3/Caspase-3 was significantly downregulated (*p <* 0.01) and the expression of Bcl-2/Bax was significantly upregulated (*p <* 0.05) in the 1–10 μg/mL Erzhi formula group compared with that in the DEX group (Figures 2C, D). In addition, the expression of Cleaved caspase-3/Caspase-3 and Bcl-2/Bax in the 10 μg/mL Erzhi formula group was similar to that of the control group.

### 3.3 The Erzhi formula improves DEX-induced primary cortical neuronal synaptic damage

**FIGURE 3:**
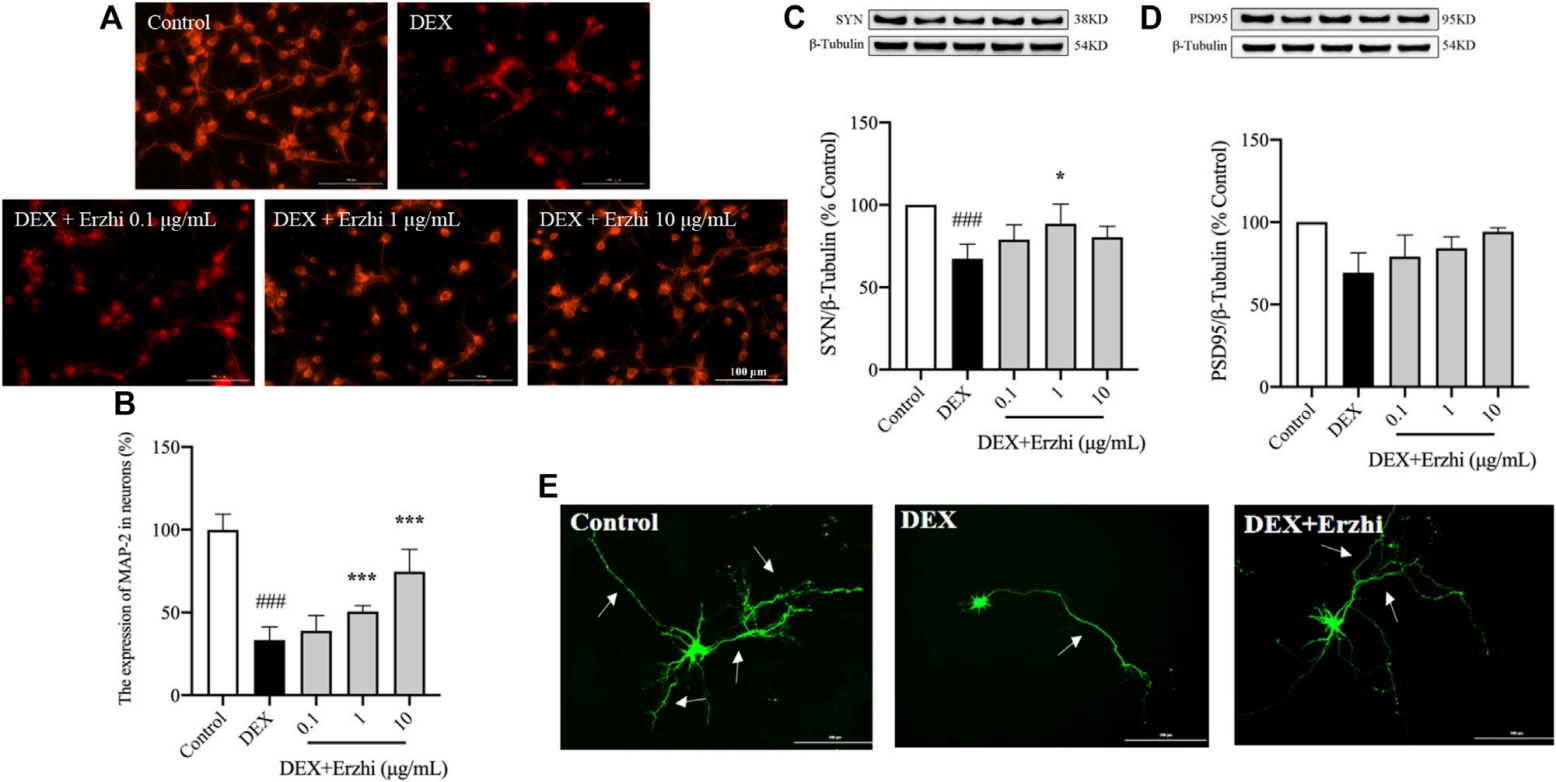
Effects of the Erzhi Formula on synapses in primary cortical neurons. **(A, B)** Fluorescent staining (bar = 100 μm) and quantitative analysis of MAP-2 (n = 3 per group). The application of the Erzhi formula could reverse the dexamethasone-induced attenuation of the MAP-2 red fluorescent signal (*p <* 0.001); **(C, D)** protein expression of SYN and PSD95 (n = 3 per group). The application of the Erzhi formula reversed the dexamethasone-induced decrease in SYN and PSD95 expression (*p >* 0.05); **(E)** synaptic morphology of primary cortical neurons (bar = 200 μm). The Erzhi formula could improve DEX-induced dendritic damage in neurons. The results are expressed as mean ± SD. ^###^ *p* < 0.001 vs. control; ^*^ *p* < 0.05, ^***^ *p* < 0.001 vs. DEX. MAP, microtubule associated protein.

MAP-2 staining showed that the synapses of primary cortical neurons in the control group were interconnected into a network, with a strong red fluorescence signal in the cytoplasm and synapses (Figure 3A). Compared with the control group, the distribution of neurons in the DEX group was sparser, synaptic damage was severe and the red fluorescence signal was significantly weaker (*p <* 0.001); compared with the DEX group, the red fluorescence signal in the 1–10 μg/mL Erzhi formula group was significantly enhanced (*p <* 0.001) (Figure 3B). In the statistical analysis of fluorescence intensity, the reduction of MAP-2 expression in neuronal cells induced by 10^–6^ mol/L DEX was 33% compared to the control group, while the reduction of MAP-2 fluorescence intensity in neurons was reversed after the administration of the Erzhi formula, and the expression of MAP-2 was recovered to approximately 75% in the 10 μg/mL Erzhi formula group. Compared with the control group, expression of SYN and PSD95 was significantly downregulated in the DEX group (*p <* 0.001); compared with the DEX group, 1 μg/mL Erzhi formula significantly upregulated SYN expression (*p <* 0.01), and 0.1–10 μg/mL Erzhi formula had the potential to upregulate PSD95 expression (*p >* 0.05) (Figures 3C, D). Calcium phosphate transfection revealed that the control group had a complete cellular structure and abundant protrusions. Compared with the control group, the DEX group had shriveled cellular structures and significantly reduced dendrites; the Erzhi formula could improve DEX-induced dendritic damage in neurons (Figure 3E).

### 3.4 Network analysis of the Erzhi formula

**FIGURE 4:**
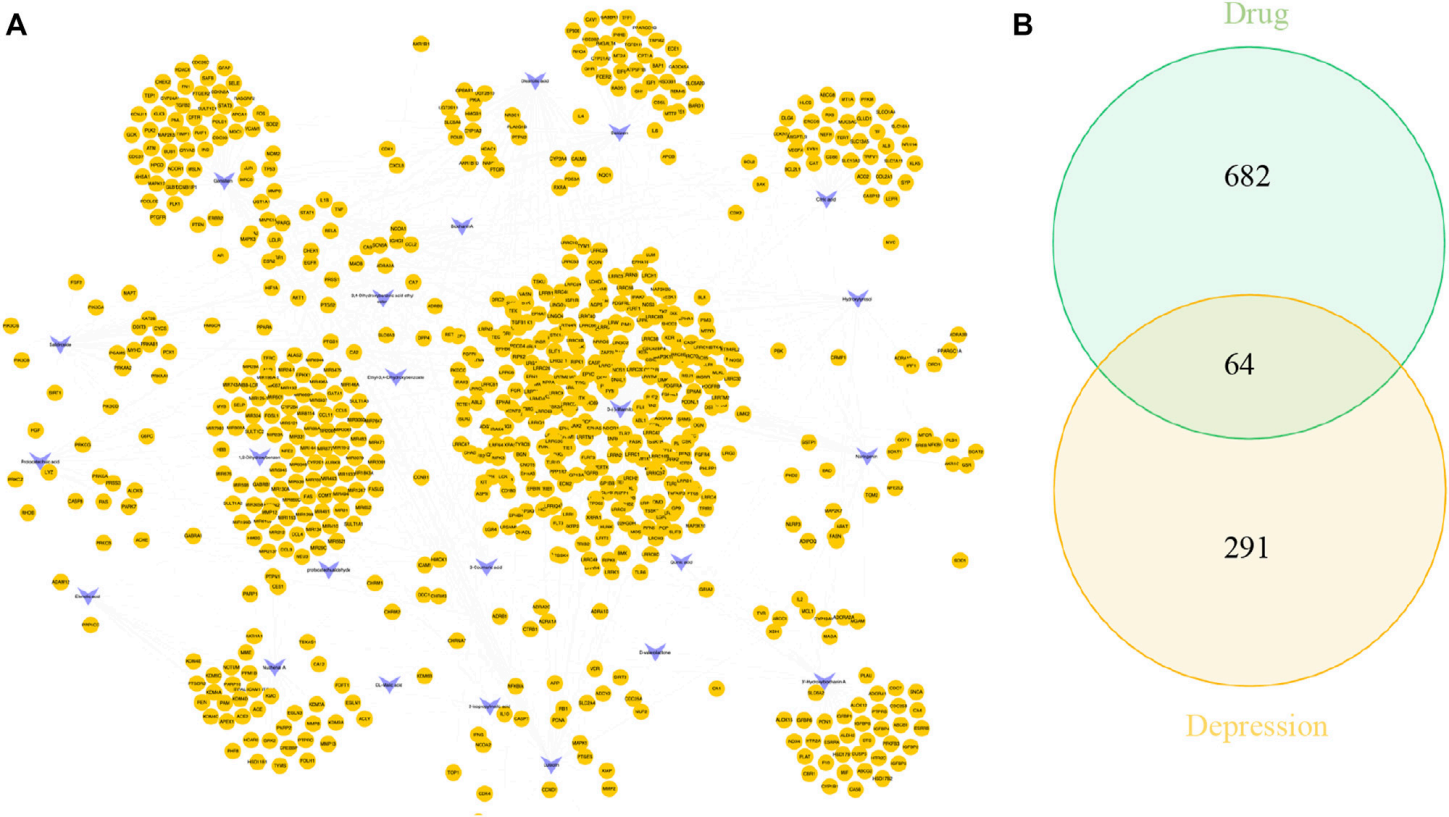
Network pharmacological analysis of the Erzhi Formula for treatment of depression. **(A)** The active ingredients of the Erzhi Formula and their corresponding targets; **(B)** the number of potential targets of the Erzhi formula for the treatment.

**FIGURE 5:**
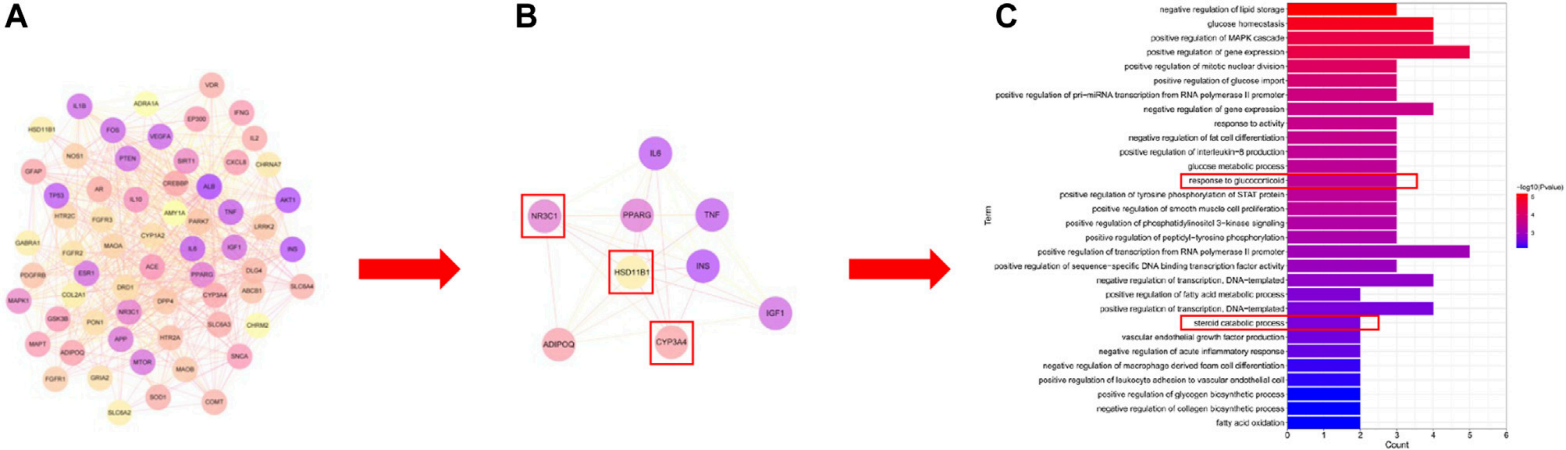
Network pharmacological analysis of the Erzhi Formula for treatment of depression. **(A)** PPI analysis of the 64 potential therapeutic targets; **(B)** cluster analysis of the 64 targets yielded nine steroid metabolism-related targets; **(C)** GO gene function enrichment analysis. PPI, protein-protein interaction.

UHPLC/Q-Orbitrap MS analysis was used to analyze rat plasma samples after oral administration of the Erzhi formula extract, and a total of 64 chemical components were identified (unpublished data from the research group). The 64 chemical components in the Erzhi formula that are absorbed into blood were analyzed using the Discovery Studio™ software with the “lter by Lipinski and Veber Rules” and 23 active ingredients were obtained (Supplementary Table S1), including 3′-Hydroxybiochanin A, Biochanin-A, and Salidroside. The CTD, HERB, and SwisstargetPrediction databases were used to search and predict the 23 active ingredients corresponding to 746 potential targets. The targets of active ingredients and potential targets were imported into Cytoscape 3.7.1 software to obtain the “active ingredient-target” network diagram (Figure 4A). In the diagram, the light purple irregular shapes represent the 23 active ingredients, the yellow circles in the middle represent the 746 potential targets, and the lines in the middle represent the interrelationship between the active ingredients and the targets. For example, target Bcl-2 could be regulated by citric acid, genistein, naringenin, protocatechuic acid, and salidroside, target SYN1 could be regulated by citric acid, target HSD11B1 (11β-HSD1) could be regulated by nuzhenal A, and NR3C1 could be regulated by oleanoic acid. By searching the GENECARDS database for depression, 3,170 depression disease targets were obtained, and 355 targets were obtained with a “relevance score >5″as the reference value. The PPI of depression was obtained (Supplementary Figure S1A) by analyzing the STRING database and Cytoscape 3.7.1 software. The enrichment analysis of gene function types (Supplementary Figure S1B) revealed that the 5-Hydroxytryptamine receptor family, steroid hormone receptor, and dopamine receptor family were regulated. The 746 active ingredient potential targets were intersected with 355 depression disease targets after screening using R language software, and 64 core targets for the Erzhi formula to treat depression were obtained (Figure 4B). The 64 core targets were analyzed in the STRING database, and the PPI network was visualized using Cytoscape 3.7.1 software (Figure 5A), with the target color changes representing the relative magnitude of the degree values. Subsequent clustering analysis allowed for the extraction of nine targets related to steroid metabolism (Figure 5B). The nine core targets obtained from the clustering analysis were analyzed for GO functions, and a total of 38 functions with *p*-value <0.01 was obtained. The top 30 functions were selected in descending order of *p*-value for display (Figure 5C), where the vertical coordinates represent the function names, and the horizontal coordinates indicate the number of targets involved. The functions closely related to our study were steroid catabolic processes (*p*-value = 2.49E-03) and response to glucocorticoids (*p*-value = 3.39E-04), and the targets included HSD11B1, CYP3A4, and NR3C1, amongst others.

The Erzhi formula influences 11β-HSD1 activity *in vitro* and affects the 11β-HSD1-GC/glucocorticoid receptor (GR) signaling pathway in primary cortical neurons.

**FIGURE 6:**
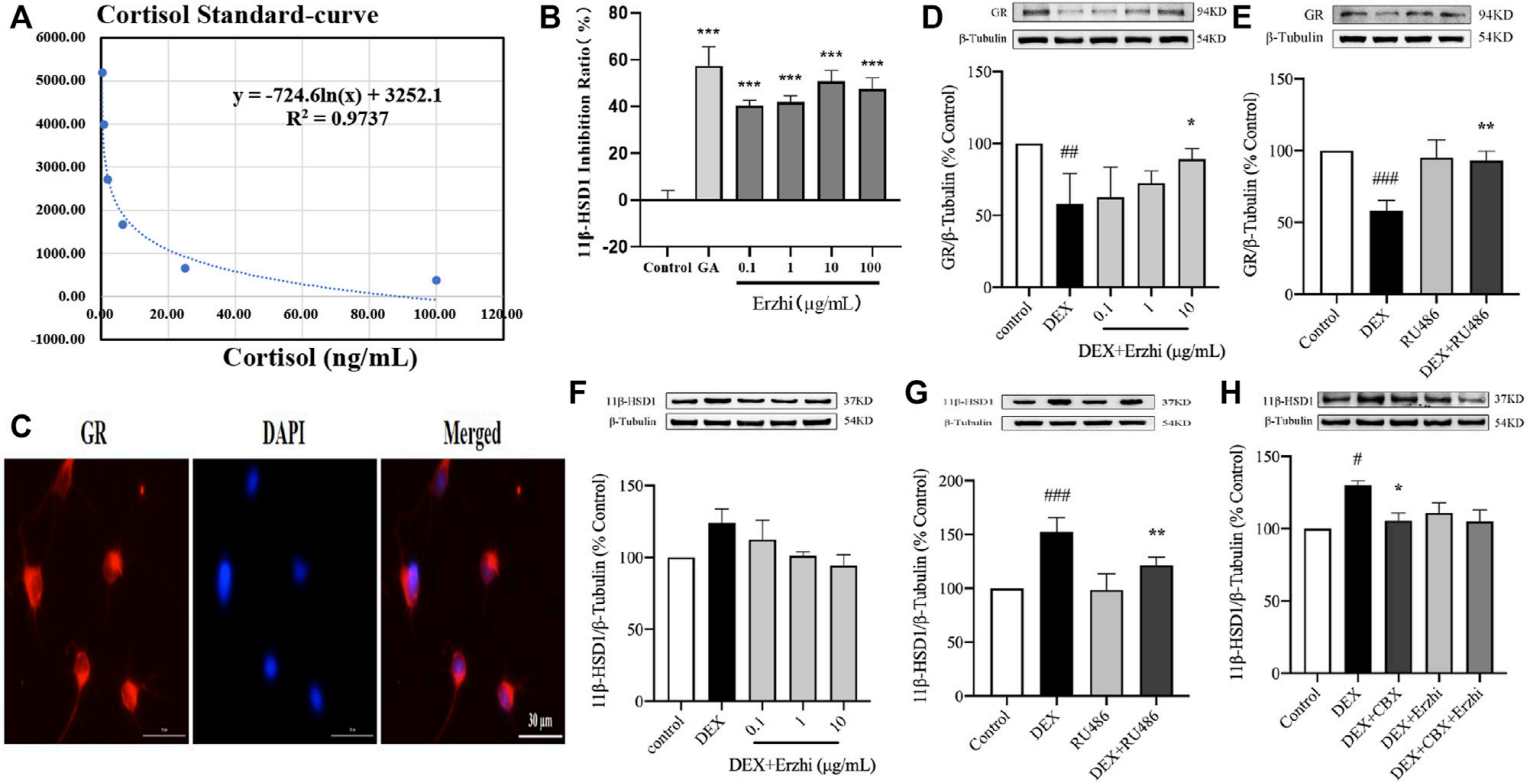
Effect of the Erzhi formula on 11β-HSD1. **(A, B)** Effect of the Erzhi formula on 11β-HSD1 activity by the HTRF method *in vitro* (n = 3 per group). 0.1–100 μg/mL Erzhi formula could significantly inhibit hepatic microsomal 11β-HSD1 activity (*p <* 0.001); **(C)** identification of GR in primary cortical neurons (bar = 30 μm); **(D–H)** protein expression of GR and 11β-HSD1 (n = 3 per group). RU486, CBX, and the Erzhi formula could affect the expression of GR and 11β-HSD1. The results are expressed as mean ± SD. ^###^ *p* < 0.001 vs. control; ^*^ *p* < 0.05, ^***^ *p* < 0.001 vs. DEX. ^##^ *p* < 0.01, ^###^ *p* < 0.001 vs. control; ^*^ *p* < 0.05, ^**^ *p* < 0.01, ^***^ *p* < 0.001 vs. DEX.

Compared to the control group, using the HTRF assay, 0.1–100 μg/mL Erzhi formula showed significant inhibition of hepatic microsomal 11β-HSD1 activity (*p <* 0.001) (Figures 6A, B), where glycyrrhetinic acid (GA) had an inhibition efficiency of approximately 58% and 0.1, 1, 10, and 100 μg/mL Erzhi formula had inhibition efficiencies of 40%, 42%, 50%, and 48%, respectively. Microscopic observations showed that GR-positive cells emitted red fluorescence, indicating that GR was widely expressed in the cytoplasm of primary cortical neurons (Figure 6C). Compared with the control group, GR expression was significantly reduced by half in the DEX group (*p* < 0.01). Compared with the DEX group, 10 μg/mL Erzhi formula significantly reversed GR expression (*p* < 0.05) (Figure 6D). After administration of 1 μM GR blocker RU486, there was no statistical difference in the RU486 group (*p >* 0.05); compared to the DEX group, RU486 significantly upregulated GR expression (*p <* 0.01) (Figure 6E). Compared to the control group, DEX upregulated 11β-HSD1 expression (*p <* 0.001) and 0.1–10 μg/mL Erzhi formula upregulated 11β-HSD1 expression (*p >* 0.05) (Figure 6F). Compared to the DEX group, RU486 significantly downregulated 11β-HSD1 expression (*p <* 0.01) (Figure 6G). After administration of the 11β-HSD1 inhibitor CBX, the expression of 11β-HSD1 was significantly downregulated (*p <* 0.05) (Figure 6H).

## 4 Discussion

GC is important for maintaining the stability of the internal environment under stress, and is an immunosuppressant involved in regulating various metabolic pathways in the body and the function of the central nervous system (Moraitis et al., 2016). However, under stressful conditions, excess GC affects not only the functioning of the cardiovascular and peripheral immune systems but also the central nervous system by inducing psychiatric disorders such as depression or cognitive impairment (Joëls, 2018). DEX is a synthetic GC with anti-inflammatory and immunosuppressive effects (Parra-Sanchez and Parada, 2013), as well as a stress hormone with receptors widely distributed in the central nervous system.

Excess GC secreted under stress binds to GR in the cytoplasm and translocates to the nucleus, initiating or modifying gene transcription, causing apoptosis and synaptic damage, and ultimately damaging the central nervous system. Apoptosis is a physiological process that occurs in the body to maintain its own stability (Xia et al., 2016), and Bcl-2 and Bax act synergistically to regulate apoptosis (Noguti et al., 2015). Bcl-2 exerts its anti-apoptotic effects mainly by inhibiting Caspase-3 activation, blocking cytochrome C release, and maintaining intracellular calcium homeostasis (Jianyu et al., 2019). Stress stimuli can lead to the cleavage and activation of Caspase-3 precursors to form cleaved Caspase-3, which can degrade structural and functional proteins in cells and thus initiate the apoptotic process (Wang et al., 2019). Our results showed that DEX-induced apoptosis in primary cortical neurons was reduced by upregulating Bcl-2/Bax and downregulating cleaved Caspase-3/Caspase-3 protein expression. However, application of the Erzhi formula can downregulate Bcl-2/Bax and upregulate cleaved Caspase-3/Caspase-3 protein expression to exert anti-apoptotic effects.

Synaptic integrity is essential for maintaining normal neuronal function. Studies have shown that high concentrations of GC induce synaptic damage in the neurons. MAP-2 is a neuron-specific cytoskeletal protein involved in dendrite formation and maintenance of neuronal function (Kim et al., 2020). SYN and PSD95 are synaptic plasticity-associated proteins and markers of neuronal synaptic damage. PSD95 is involved in regulating synaptic strength, plasticity, maturation, and the promotion of dendritic spine morphogenesis and maturation (Ma et al., 2017), while SYN is mainly located at the presynaptic terminal and is involved in the regulation of synaptic vesicle cytokinesis. SYN deficiency may lead to synaptic dysfunction (Somayaji et al., 2020; Somayaji et al., 2021). Calcium phosphate precipitation is the preferred method for the transfection of appressed cells, and GFP transfection of neurons can clearly delineate the axons and dendrites of individual neurons. Our results showed that GFP was stably expressed in primary neurons, and the Erzhi formula ameliorated DEX-induced dendritic damage in primary cortical neurons. The Erzhi formula can improve neural growth and alleviate synaptic damage by regulating synaptic-related protein expression.

To investigate the mechanism of the Erzhi formula, we conducted a network pharmacological exploration. We found that the active ingredients in the Erzhi formula included 3′-Hydroxybiochanin A, Biochanin-A, Citric acid, Daidzein, and Salidroside. Potential targets included SYN, CASP3, CASP9, IL6, IL10, and so on. To elucidate the role of the Erzhi formula in treating depression, we crossed the potential targets of the Erzhi formula with the targets of depression and identified 64 intersecting targets. Nine core targets were selected using PPI and cluster analyses. They were found to be involved in steroid metabolic function after GO gene function enrichment analysis, which is consistent with the regulation of steroid hormone receptors in PPI analysis of depression. Among steroid metabolic functions, the target 11β-HSD1 is particularly important. 11β-HSD1 is a major determinant of cortisol overload, and its inhibition alleviates metabolic abnormalities (Mohd Azmi et al., 2021). Cortisol status is an objective marker of the stress response and the end-product of the HPA AXIS in response to various stressors (Qin et al., 2016; Huang et al., 2020). Neurosteroid hormone action can alter neuronal excitability and modulate stressful emotional responses such as anxiety and depression (Mendell and MacLusky, 2018). Depression leads to abnormalities in the HPA AXIS and abnormally high levels of corticosterone, which binds to corticosteroid receptors in the brain and induces various impairments (Vincent et al., 2013).

RU486, a GR blocker, has a high affinity for GR and antagonizes the binding of GC to GR by competitive occupation (Zalachoras et al., 2013). RU486 not only reverses the reduction in hippocampal neurons induced by long-term GC application but also reduces stress-induced apoptosis of newborn neurons and improves cognitive impairment (Mayer et al., 2006; Llorens-Martín and Trejo, 2011). GR mediates the action of GC by activating genes through association with the glucocorticoid responsive elements (GRE) (Timmermans et al., 2019). Sequence analysis of the cloned 11β-HSD1 gene revealed the presence of a CTGAT-ACAG sequence similar to the structural sequence of GRE at base pairs 197 to 190 upstream of the transcription start site of the 11β-HSD1 gene. Therefore, we hypothesized that DEX might regulate the gene and protein expression of 11β-HSD1 through GR-induced 11β-HSD1 gene promoter activation. Our results showed that RU486 blocked DEX-induced 11β-HSD1 increase in primary cortical neurons, suggesting that 11β-HSD1 expression caused by GC action on primary cortical neurons is partially mediated by GR. CBX, an inhibitor of 11β-HSD1, inhibits the activity of 11β-HSD1 and protects cells from neurotoxic damage (Sharma et al., 2021). 11β-HSD1, an isoform of 11β-HSD (Kadmiel and Cidlowaki, 2013), is a low-affinity NADP^+−^dependent enzyme that catalyzes the interconversion between hydrocortisone and cortisone (Lagos et al., 2014). It is widely distributed in GC target organs, such as the liver, brain, and adipose (Bisschop et al., 2013). 11β-HSD1 is an important enzyme that regulates peripheral GC metabolism. Under stress, GC promotes hepatic 11β-HSD1 gene and protein expression and activity, and excess cortisol accelerates the negative feedback failure of the HPA AXIS, further exacerbating stress injury (Hinkelmann et al., 2009; Sarabdjitsingh et al., 2014). In our study, we selected liver microsomes and used CORT as the substrate and GA as the positive control drug to determine the concentration of cortisol using the HTRF method and found that the Erzhi formula had a significant effect on hepatic microsomal 11β-HSD1 activity *in vitro*. The Erzhi formula regulates GR and 11β-HSD1 protein expression in primary cortical neurons.

According to the above results, the Erzhi formula may exert an anti-depression effect by reducing neural apoptosis and alleviating synaptic damage. However, only network analysis and *in vitro* experiments were performed in the present study, and further *in vivo* analyses are required to confirm these results. Notably, the network analysis revealed a correlation between the modulation of steroid metabolism by the Erzhi formula and its treatment of depression, which was confirmed by *in vitro* validation of enzyme activity and protein expression assays, providing ideas for further studies.

## 5 Conclusion

In summary, the Erzhi formula has been shown to be effective in improving DEX-induced damage to primary cortical neurons, primarily by reducing neuronal apoptosis and improving synaptic damage. This potential mechanism is partly related to the inhibition of the 11β-HSD1-GC/GR signaling pathway.

## Data Availability

The datasets presented in this study can be found in online repositories. The names of the repository/repositories and accession number(s) can be found in the article/Supplementary Material.
