# Supplementary material for: Protective effects and mechanisms of the Erzhi formula on glucocorticoid induced primary cortical neuron injury
Source: Front Pharmacol. 2023 Feb 27;14:1038492. doi: 10.3389/fphar.2023.1038492 (PMC10008893; doi:10.3389/fphar.2023.1038492)
Supplement: Supplementary file 1 [file DataSheet1.docx]

Supplementary Material

# Supplementary Table 1

| Ingredients | Molecular Formula |
| --- | --- |
| 1,2-Dihydroxybenzen | C_10_H_12_O_2_ |
| 2-isopropylmalic acid | C_7_H_12_O_5_ |
| 3,4-Dihydroxybenzoic acid | C_9_H_10_O_4_ |
| 3-Coumaric acid | C_9_H_8_O_3_ |
| 3'-Hydroxybiochanin A | C_16_H_12_O_6_ |
| Biochanin-A | C_22_H_22_O_10_ |
| Citric acid | C_6_H_8_O_7_ |
| D-(-)-Mannitol | C_6_H_14_O_6_ |
| Daidzein | C_15_H_10_O_4_ |
| DL-Malic acid | C_4_H_6_O_5_ |
| D-valerolactone | C_5_H_8_O_2_ |
| Elenolic acid | C_11_H_14_O_6_ |
| Ethyl-3,4-Dihydroxybenzoate | C_9_H_10_O_4_ |
| Genistein | C_15_H_10_O_5_ |
| Hydroxytyrosol | C_8_H_10_O_3_ |
| Luteolin | C_15_H_10_O_6_ |
| Naringenin | C_15_H_12_O_5_ |
| Nuzhenal A | C_10_H_14_O_5_ |
| Oleanolic acid | C_30_H_4_8O_3_ |
| protocatechualdehyde | C_7_H_6_O_3_ |
| Protocatechuic acid | C_7_H_6_O_4_ |
| Quinic acid | C_7_H_12_O_6_ |
| Salidroside | C_14_H_20_O_7_ |

# Supplementary Figure 1


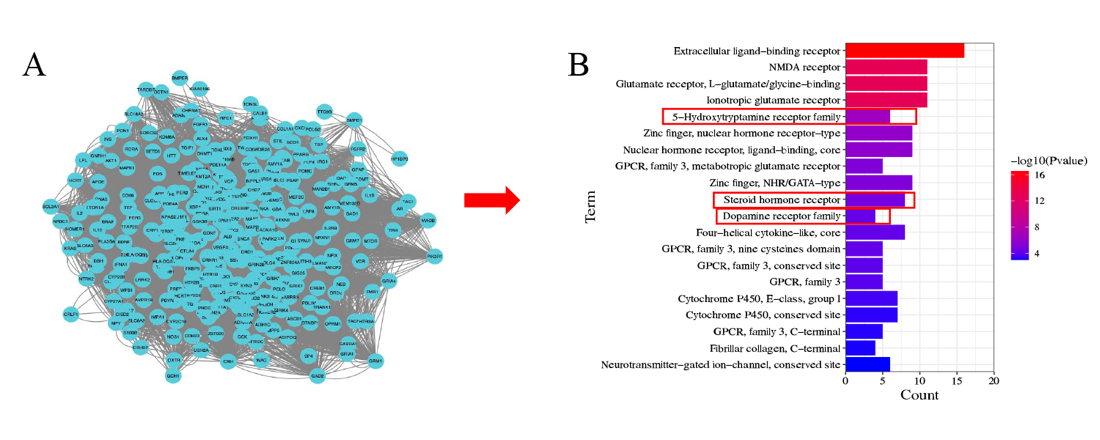


**Supplementary Figure 1.** (A)The PPI analysis of depression targets (Relevance score > 5). (B) GO gene function enrichment analysis
